# Factors Associated With Humeral Avulsion of Glenohumeral Ligament Lesions in Patients With Anterior Shoulder Instability: An Analysis of the MOON Shoulder Instability Cohort

**DOI:** 10.1177/23259671231206757

**Published:** 2023-10-27

**Authors:** Ryan D. Freshman, Alan L. Zhang, C. Benjamin Ma, Brian T. Feeley, Shannon Ortiz, Jhillika Patel, Warren Dunn, Brian R. Wolf, Carolyn Hettrich, Drew Lansdown, MOON Shoulder Group collaborators, Keith M. Baumgarten, Julie Y. Bishop, Matthew J. Bollier, Robert H. Brophy, Jonathan T. Bravman, Charles L. Cox, Gregory L. Cvetanovich, John A. Grant, Rachel M. Frank, Grant L. Jones, John E. Kuhn, Scott D. Mair, Robert G. Marx, Eric C. McCarty, Bruce S. Miller, Adam J. Seidl, Matthew V. Smith, Rick W. Wright

**Affiliations:** Department of Orthopedic Surgery, University of California–San Francisco, San Francisco, California, USA; Department of Orthopedic Surgery, University of California–San Francisco, San Francisco, California, USA; Department of Orthopedic Surgery, University of California–San Francisco, San Francisco, California, USA; Department of Orthopedic Surgery, University of California–San Francisco, San Francisco, California, USA; University of Iowa, Iowa City, Iowa, USA; Brigham and Women’s Hospital, Boston, Massachusetts, USA; Fondren Orthopedic Group, Houston, Texas, USA; Department of Orthopedic Surgery, University of California–San Francisco, San Francisco, California, USA; Brigham and Women’s Hospital, Boston, Massachusetts, USA; Department of Orthopedic Surgery, University of California–San Francisco, San Francisco, California, USA; Orthopedic Institute of Sioux Falls, Sioux Falls, South Dakota, USA; The Ohio State University Wexner Medical Center, Columbus, Ohio, USA; University of Iowa, Iowa City, Iowa, USA; Washington University, St Louis, Missouri, USA; University of Colorado School of Medicine, Aurora, Colorado, USA; Vanderbilt University Medical Center, Nashville, Tennessee, USA; The Ohio State University Wexner Medical Center, Columbus, Ohio, USA; MedSport, University of Michigan, Ann Arbor, Michigan, USA; University of Colorado School of Medicine, Aurora, Colorado, USA; The Ohio State University Wexner Medical Center, Columbus, Ohio, USA; Vanderbilt University Medical Center, Nashville, Tennessee, USA; University of Kentucky, Lexington, Kentucky, USA; Hospital for Special Surgery, New York, New York, USA; University of Colorado School of Medicine, Aurora, Colorado, USA; MedSport, University of Michigan, Ann Arbor, Michigan, USA; University of Colorado School of Medicine, Aurora, Colorado, USA; Washington University, St Louis, Missouri, USA; Vanderbilt University Medical Center, Nashville, Tennessee, USA; Investigation performed at the University of California–San Francisco, San Francisco, California, USA

**Keywords:** humeral avulsion of the glenohumeral ligament, magnetic resonance imaging, risk factors, shoulder instability

## Abstract

**Background:**

Humeral avulsion of the glenohumeral ligament (HAGL) lesions are an uncommon cause of anterior glenohumeral instability and may occur in isolation or combination with other pathologies. As HAGL lesions are difficult to detect via magnetic resonance imaging (MRI) and arthroscopy, they can remain unrecognized and result in continued glenohumeral instability.

**Purpose:**

To compare patients with anterior shoulder instability from a large multicenter cohort with and without a diagnosis of a HAGL lesion and identify preoperative physical examination findings, patient-reported outcomes, imaging findings, and surgical management trends associated with HAGL lesions.

**Study Design:**

Cross-sectional study; Level of evidence, 3.

**Methods:**

Patients with anterior glenohumeral instability who underwent surgical management between 2012 and 2020 at 11 orthopaedic centers were enrolled. Patients with HAGL lesions identified intraoperatively were compared with patients without HAGL lesions. Preoperative characteristics, physical examinations, imaging findings, intraoperative findings, and surgical procedures were collected. The Student *t* test, Kruskal-Wallis *H* test, Fisher exact test, and chi-square test were used to compare groups.

**Results:**

A total of 21 HAGL lesions were identified in 915 (2.3%) patients; approximately one-third (28.6%) of all lesions were visualized intraoperatively but not identified on preoperative MRI. Baseline characteristics did not differ between study cohorts. Compared with non-HAGL patients, HAGL patients were less likely to have a Hill-Sachs lesion (54.7% vs 28.6%; *P* = .03) or an anterior labral tear (87.2% vs 66.7%; *P* = .01) on preoperative MRI and demonstrated increased external rotation when their affected arm was positioned at 90° of abduction (85° vs 90°; *P* = .03). Additionally, HAGL lesions were independently associated with an increased risk of undergoing an open stabilization surgery (odds ratio, 74.6 [95% CI, 25.2-221.1]; *P* < .001).

**Conclusion:**

Approximately one-third of HAGL lesions were missed on preoperative MRI. HAGL patients were less likely to exhibit preoperative imaging findings associated with anterior shoulder instability, such as Hill-Sachs lesions or anterior labral pathology. These patients underwent open procedures more frequently than patients without HAGL lesions.

Anterior glenohumeral instability after a traumatic shoulder dislocation is a commonly encountered condition, with a yearly incidence of approximately 1.7% in the general population.^12^ Recurrent dislocation events occur frequently, especially in younger athletic patients, and can represent a significant obstacle that prevents return to sports or work. While a variety of intra-articular injuries can occur in patients with anterior glenohumeral instability, up to 90% of patients have an avulsion of the anteroinferior glenoid labrum,^24^ also known as a Bankart lesion.

Another less common but clinically relevant injury associated with anterior glenohumeral instability is a humeral avulsion of the glenohumeral ligaments (HAGL) lesion. These injuries typically occur through a hyperabduction/external rotation mechanism^20^ and result in tearing the anterior band of the inferior glenohumeral ligament (IGHL), a primary static stabilizer of the shoulder. HAGL lesions are estimated to occur in approximately 2% to 9% of patients with recurrent shoulder instability. However, the epidemiological understanding of these lesions is largely confined to reports on smaller cohorts of patients.^5,18^ These injuries may occur in isolation or combination with other pathologies and are difficult to detect via preoperative magnetic resonance imaging (MRI).^5,18,21^ Thus, they have the potential to remain unrecognized and may result in continued glenohumeral instability if not properly addressed at the time of surgical stabilization. Although previous studies have defined patient characteristics and concomitant injury profiles associated with HAGL lesions,^3,31^ few studies have identified specific demographic variables or injury factors that may predispose patients to sustaining a HAGL lesion. The influence of HAGL lesions on preoperative patient-reported outcomes (PROs) and surgical decision making is unclear.

This study aimed to describe the epidemiology of HAGL lesions in a large, multicenter population of patients undergoing surgical treatment for shoulder instability. We sought to identify patient-related factors associated with a HAGL lesion and assess the effect of HAGL lesions on preoperative physical examinations, PROs, imaging findings, and surgical procedures performed. We hypothesized that patients with HAGL lesions would show increased shoulder range of motion (ROM) on the physical examination and would have increased rates of associated intra-articular shoulder pathology such as Bankart lesions.

## Methods

The study protocol received institutional review board approval. Patients who underwent primary shoulder stabilization between 2012 and 2020 for anterior shoulder instability provided informed consent and were prospectively enrolled in the Multicenter Orthopedic Outcomes Network (MOON) Shoulder Group. The MOON Shoulder Group comprises 11 orthopaedic centers in the United States, including academic and private practice settings. The MOON Shoulder Group prospectively collects a variety of orthopaedic outcome data from patients with shoulder instability.^15^

**Figure 1.**
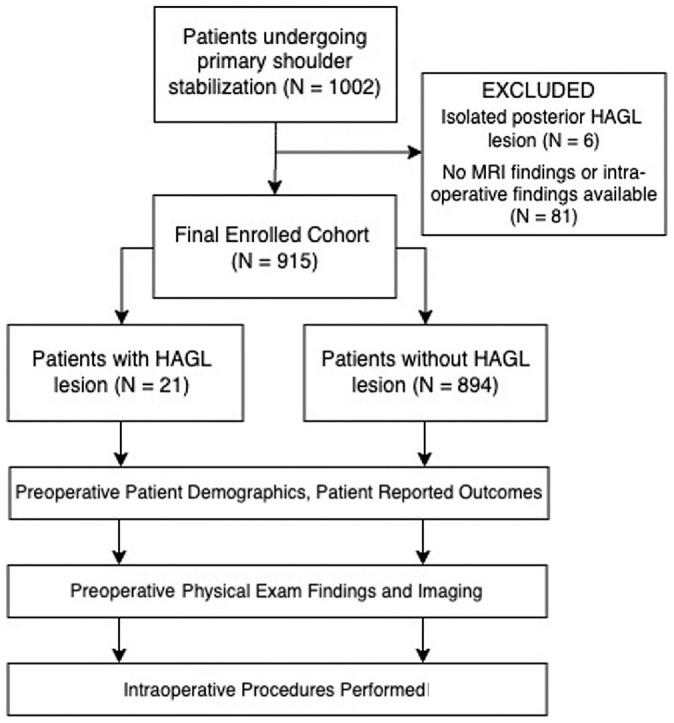
CONSORT diagram outlining patient cohort allocations. CONSORT, Consolidated Standards of Reporting Trials; HAGL, humeral avulsion of the glenohumeral ligaments; MRI, magnetic resonance imaging.

Patients with a HAGL lesion diagnosed via arthroscopy and patients without a HAGL lesion were identified as the case and control cohorts, respectively. Patients whose MRI or arthroscopic findings were not documented or who had a diagnosed isolated posterior HAGL lesion were excluded from the analysis. Complete patient cohort allocation is outlined in Figure 1.

Preoperative data collected included demographic characteristics, sports participation, number of previous shoulder dislocations, and duration of instability symptoms. In addition, preoperative PROs were collected, including the RAND 36-Item Health Survey 1.0 (including physical score and mental health score),^11^ American Shoulder and Elbow Surgeons (ASES) shoulder score,^22^ Western Ontario Shoulder Instability Index (WOSI) score,^13^ Shoulder Activity Scale (SAS) score,^4^ EuroQol 5 Dimensions (EQ-5D) score,^28^ and Single Assessment Numeric Evaluation (SANE) score.^30^ Preoperative physical examination data included shoulder strength, ROM, presence of apprehension signs, and Beighton score. Plain radiograph and MRI findings for each patient were also included; any bony defects were noted on either plain radiograph or MRI, while evidence of labral pathology was documented on MRI. Surgeons specified which type of arthroscopic and/or open surgical techniques were performed and if any ancillary procedures were performed during stabilization.

### Statistical Analyses

Demographic data, preoperative PROs, physical examination results, imaging findings, and surgical procedures were compared between the 2 groups. SPSS software Version 27 (IBM) was used for statistical analysis. The Kolmogorov-Smirnov test was utilized to assess whether continuous data were distributed normally. The Student *t* test was used to compare normally distributed continuous data, while the Mann-Whitney *U* test was used to compare nonnormally distributed continuous data. Categorical data were compared using the chi-square or Fisher exact test, and ranked ordinal data were compared using Kruskal-Wallis *H* testing. Logistic regression analysis was also performed to determine the independent predictors of patients needing to undergo an open stabilization procedure. Variables entered into the regression model included common demographic variables, variables that are typically considered by surgeons when evaluating patients with shoulder instability, or variables found to have statistical significance on univariate analysis. Significance for all tests was set at *P* < .05.

## Results

### Patient Characteristics

During enrollment, 915 patients underwent an arthroscopic, open, or combined procedure for recurrent anterior shoulder instability. A total of 21 patients were diagnosed with a HAGL lesion intraoperatively, with a prevalence of 2.3%. There were no differences between patients with and without a HAGL lesion with regard to mean age, mean body mass index (BMI), distribution of male and female patients, smoking prevalence, duration of instability symptoms, number of lifetime dislocations, or dislocations in the year leading up to surgery (Table 1). Among the patients with a HAGL lesion, 66.7% sustained a shoulder dislocation while playing sports, compared with 72.7% of patients without a HAGL lesion (*P* = .63).

**Table 1:** Baseline Characteristics of the Study Cohorts^*a*^

Characteristic	HAGL (n = 21)	No HAGL (n = 894)	*P*
Age, y	27.2 ± 12	24.7 ± 9.2	.23
Female sex, %	28.6	21.3	.42
BMI, kg/m^2^	24.3 ± 4.5	25.3 ± 4.4	.31
Smoking, %	4.8	4.1	.59
Lifetime dislocations, %			.80
0^*b*^	6.7	14.6	
1	13.3	19	
2-5	66.7	39.8	
>5	13.4	26.5	
Dislocations in past year, %			.53
0	28.6	16.1	
1	14.3	24.7	
2-5	38.1	38	
>5	19	21.1	
Duration of symptoms, %			.10
<1 mo	4.8	8.9	
1-3 mo	14.3	19	
4-6 mo	4.8	13.9	
7-12 mo	9.5	11	
>1 y	66.7	47.2	

a Data are reported as mean ± SD unless otherwise indicated. BMI, body mass index; HAGL, humeral avulsion of the glenohumeral ligaments.

b Indicates subjective symptoms or subluxations only.

### Preoperative Imaging

Preoperative MRI correctly identified 15 of 21 HAGL lesions, while the remaining 6 were not identified before surgery and only discovered intraoperatively. Using arthroscopic diagnosis of a HAGL lesion as the gold standard, the calculated sensitivity and specificity of MRI for detecting a HAGL lesion were 71.4% and 99.2%, respectively. The calculated positive and negative predictive values of MRI were 67.8% and 99.3%, respectively.

**Figure 2.**
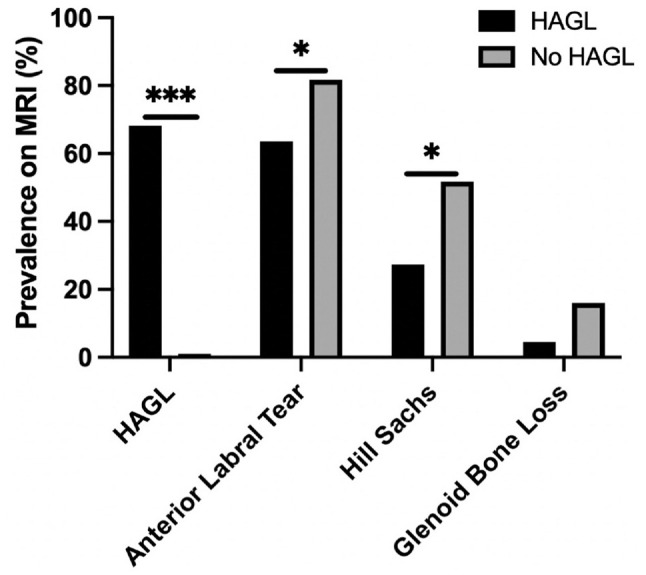
Prevalence of preoperative MRI findings classically associated with anterior shoulder instability. HAGL, humeral avulsion of the glenohumeral ligaments; MRI, magnetic resonance imaging. *P < .05, ***P < .001.

Compared with patients without HAGL lesions, patients with HAGL lesions were less likely to be diagnosed with a Hill-Sachs lesion (54.7% vs 28.6%; *P* = .03) or an anterior labral tear (87.2% vs 66.7%; *P* = .01) via MRI (Figure 2 and Appendix Table A1). There was no difference in the prevalence of glenoid bone loss (0% vs 12.1%; *P* = .16) or humeral defects (13.6% vs 23.9%; *P* = .24) observed on preoperative plain radiographs between the HAGL and non-HAGL groups. Additionally, no significant difference in the prevalence of anterior glenoid bone loss was seen on MRI between the 2 groups (4.8% vs 15.1%; *P* = .35).

### Preoperative PROs and Physical Examination

There were no significant differences in the RAND-36 physical score or mental health score, ASES shoulder score, WOSI score, SAS score, EQ-5D score, or SANE score between patients with and without HAGL lesions (Table 2).

**Table 2:** Preoperative Patient-Reported Outcomes^*a*^

Scores	HAGL	No HAGL	*P*
ASES	73.1 ± 16.3	67 ± 20.2	.17
EQ-5D	75.5 ± 15.5	75.1 ± 16.9	.93
RAND-36 mental	51.6 ± 10.3	50.3 ± 10.4	.59
RAND-36 physical	46.9 ± 9.2	47.1 ± 8	.89
WOSI	45.7 ± 17	43 ± 19.5	.54
SAS	12.8 ± 4.4	13 ± 4.4	.88
SANE	46.3 ± 27.6	46.1 ± 23.8	.97

a Data are reported as mean ± SD. ASES, American Shoulder and Elbow Surgeons; EQ-5D, EuroQol 5 Dimensions; HAGL, humeral avulsion of the glenohumeral ligaments; SANE, Single Assessment Numeric Evaluation; SAS, Shoulder Activity Scale; WOSI, Western Ontario Shoulder Instability Index.

Patients with a HAGL lesion demonstrated increased external rotation when their affected arm was positioned in 90° of shoulder abduction compared with non-HAGL patients (90° vs 85°; *P* = .03). However, HAGL patients did not demonstrate any significant difference in ROM between their affected and unaffected shoulders. There were no other differences between HAGL and non-HAGL patients with respect to shoulder ROM, presence of apprehension signs, or Beighton score (Table 3).

**Table 3:** Preoperative Physical Examination Findings^*a*^

Variable	HAGL	No HAGL	*P*
External rotation (90° of abduction), deg	90°± 9°	85°± 17°	.03^*b*^
Internal rotation (90° of abduction), deg	63°± 16°	59°± 18°	.33
External rotation (side), deg	68°± 14°	64°± 19°	.40
Internal rotation (side), deg	60°± 0°	58°± 6°	.12
Forward elevation, deg	170°± 22°	169°± 21°	.74
Abduction, deg	165°± 23°	163°± 26°	.81
Beighton score	1.3 ± 1.7	1 ± 2	.66
Apprehension sign present, %	90.5	88.4	≥.99

a Data are reported as mean ± SD unless otherwise indicated. HAGL, humeral avulsion of the glenohumeral ligaments.

b *P* < .05.

### Procedures Performed

There were 915 primary shoulder stabilization procedures performed within the study cohort, of which 77 were open procedures (8.4%). The breakdown of surgical procedures is outlined in Appendix Table A2.

**Figure 3.**
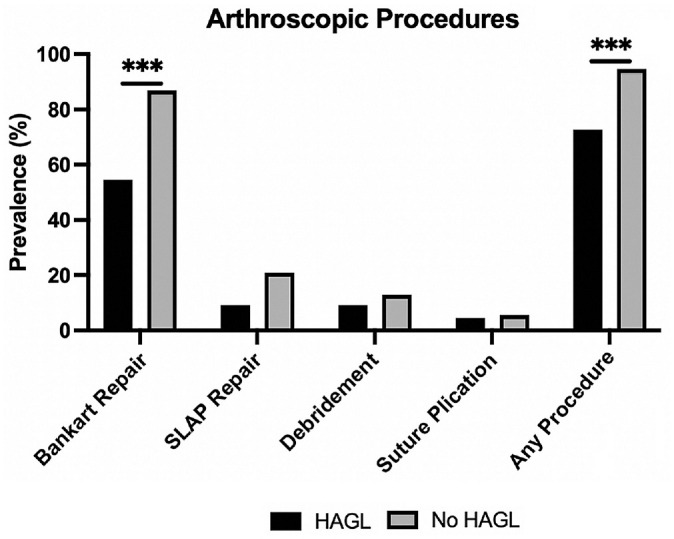
Prevalence of arthroscopic procedures performed in patients with anterior instability. HAGL, humeral avulsion of the glenohumeral ligaments; SLAP, superior labrum anterior and posterior. ***P < .001.

Arthroscopic procedures were performed in 71.4% of HAGL patients and 97.7% of non-HAGL patients (*P* < .001). Anterior Bankart repair was the most common arthroscopic procedure performed among both groups (57.1% of the HAGL patients and 90.7% of the non-HAGL group; *P* < .001) (Figure 3). With respect to other arthroscopic procedures, there were no differences in the prevalence of suture plication (*P*≥ .99 ), superior labrum anterior and posterior repair (*P* = .28), or debridement (*P* = .50) between groups.

**Figure 4.**
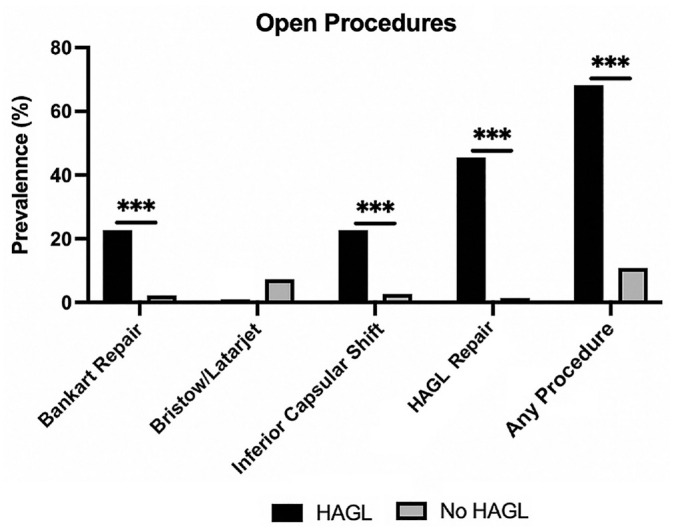
Prevalence of open surgical procedures performed in patients with anterior instability. HAGL, humeral avulsion of the glenohumeral ligaments. ***P < .001.

Open procedures were performed in 66.7% of HAGL patients and 7% of non-HAGL patients (*P* < .001). The most common open procedure performed in HAGL patients was an open HAGL repair (42.9%), while the most common open procedure performed in non-HAGL patients was a Bristow-Latarjet procedure (4.1%). HAGL patients were more likely than non-HAGL patients to undergo open Bankart repair (23.8% vs 1.4%; *P* < .001) and open inferior capsular shift (23.8% vs 2.2%; *P* < .001) (Figure 4).

Logistic regression analysis was performed to better understand the influence of multiple factors on (1) the likelihood of a HAGL lesion being present on diagnostic arthroscopy and (2) the likelihood that patients would undergo an open procedure. The model for the likelihood of a HAGL lesion being present included patient age, sex, BMI, smoking history, number of lifetime dislocations, number of dislocations in the past year, duration of instability symptoms, shoulder ROM findings on examination under anesthesia, presence versus absence of Bankart lesion and/or Hill-Sachs lesion on preoperative MRI, and glenoid bone loss noted on preoperative MRI. The model for the likelihood of undergoing an open procedure included patient age, sex, BMI, smoking history, number of dislocations in the past year, glenoid or humeral bone loss noted on preoperative MRI, and presence of a HAGL lesion as independent predictors.

The absence of a Bankart lesion (odds ratio [OR], 6.8 [95% CI, 1.4-33.9]; *P* = .01) and the absence of a Hill-Sachs lesion (OR, 6.5 [95% CI, 1.5-27.7]; *P* = .02) on preoperative MRI were independently associated with increased likelihood of a HAGL lesion being present.

HAGL lesions were independently associated with an increased likelihood of undergoing an open procedure (OR, 74.6 [95% CI, 25.2-221.1]; *P* < .001). Other independent factors associated with open procedures included a glenoid bone loss on preoperative MRI (OR, 7.6 [95% CI, 4.2-13.6]; *P* < .001) and having >5 dislocations in the year preceding surgery (OR, 3.5 [95% CI, 1.3-9.3]; *P* = .01).

## Discussion

We observed the overall prevalence of anterior HAGL lesions to be 2.3% in this large, multicenter group of patients undergoing surgical management for anterior shoulder instability. Approximately one-third of all HAGL lesions went unidentified on preoperative imaging. Patients with HAGL lesions were less likely to demonstrate additional imaging findings classically associated with anterior instability, including Hill-Sachs lesions and anterior labral pathology, and underwent open stabilization procedures more frequently than patients without HAGL lesions. Patients with HAGL lesions were also found to have small but statistically significant difference in shoulder ROM compared with patients without HAGL lesions.

HAGL lesions may be challenging to detect on preoperative advanced imaging and may be missed on diagnostic arthroscopy without proper attention to viewing the axillary pouch. In our cohort, 2.3% of patients with anterior shoulder instability were diagnosed with a HAGL lesion via diagnostic arthroscopy. While the original studies by Wolf et al^31^ and Bokor et al^2^ reported a 7.5% to 9% prevalence of HAGL lesions in a shoulder instability population, more recent works by Bui-Mansfield et al^5^ and Magee^18^ have estimated this number to be closer to 2%. One challenge in defining the true prevalence of HAGL lesions is the continued difficulty in identifying these lesions via advanced imaging, and it is possible that HAGL lesions were underreported within our cohort. We observed that approximately 30% of all HAGL lesions identified intraoperatively in our cohort were not seen on the patient’s preoperative MRI scan, and previous literature^5^ suggests this rate may be as high as 50%. There are likely various reasons for these high false-negative rates, including lack of consensus regarding imaging findings that define HAGL lesions and poorly defined specificity of HAGL-associated MRI findings such as the “J” sign. Moreover, the ability to identify HAGL lesions on MRI may be affected by the use of intra-articular contrast reagent^19^ or even the timing of MRI, with a higher prevalence of HAGL lesions noted on MRI scans that were performed within 1 week of an acute instability event compared with those performed 4 weeks later.^16^ Given the current diagnostic dilemma, further investigation is warranted to improve our ability to accurately detect HAGL lesions on preoperative MRI.

In contrast to our hypothesis, patients with HAGL lesions were found to have significantly lower rates of Hill-Sachs lesions and anterior labral tears compared with patients without HAGL lesions. To the best of our knowledge, this is the first comparative study to report this finding in a population of patients with anterior shoulder instability. Rates of concomitant intra-articular injuries associated with HAGL lesions vary throughout the literature. In a small case series of patients with HAGL lesions, Bui-Mansfield et al^5^ reported that Bankart and Hill-Sachs lesions occurred in 33% and 50% of patients, respectively. Provencher et al^27^ found that 37% of patients with HAGL lesions had concomitant labral tears, while a recent systematic review by Bozzo et al^3^ noted relatively low rates of associated Bankart lesions (15%) and Hill-Sachs lesions (13%) in this population. Although our study found higher rates of concomitant Hill-Sachs lesions and anterior labral tears in patients with HAGL lesions compared with the studies mentioned above, these rates are still lower than the historically reported prevalence of Hill-Sachs lesions and anterior labral tears in cohorts composed of patients with anterior shoulder instability.^6,24^ While the reason behind these findings is likely multifactorial, one possibility is that failure of the IGHL during a traumatic anterior shoulder dislocation reduces the subsequent contact forces between the proximal humerus and anterior-inferior glenoid rim. Although no studies have directly examined this idea in the context of HAGL lesions, Park et al^25^ found that large simulated HAGL lesions tended to shift the position of the humeral head apex, which may affect contact forces between structures in the shoulder. However, the literature on this topic remains limited, and additional investigation into the reasons underlying lower rates of anterior labral tears and Hill-Sachs lesions in HAGL patients is warranted. Nevertheless, our findings suggest that surgeons should have a heightened level of suspicion for a HAGL lesion when patients with anterior shoulder instability show no evidence of Hill-Sachs lesions or Bankart lesions.

While patients with and without HAGL lesions did not differ significantly with regard to preoperative characteristics and PROs, patients with HAGL lesions did demonstrate a small but significant increase in external rotation (90° vs 85°) at 90° of shoulder abduction. Although increased external rotation may also be observed in the presence of Bankart lesions,^8^ it has also been reported in both a case series of HAGL lesions^7^ as well as a cadaveric model of HAGL repair.^25^ These findings may be related to the role of IGHL as a restraint against excessive anterior translation and external rotation of the humerus when the arm is abducted^23^ to 90°. However, it is important to acknowledge that our findings may not be clinically significant given that our observed difference in shoulder external rotation lies below the reported^14^ minimal detectable change of 9°. While smaller HAGL lesions may not create an appreciable change in shoulder ROM, larger HAGL lesions have been shown to have greater effects on shoulder kinematics that may be clinically detectable^25,26^ and correctable after HAGL repair.^17,25,29^ We were unable to assess the size and extent of HAGL lesions within our study, which may have limited our ability to accurately detect differences in shoulder ROM because of HAGL lesions. Furthermore, it is difficult to determine whether the observed increase in external rotation was pathologic or physiologic, given that HAGL patients did not have significant differences between their affected and unaffected shoulders. Nonetheless, future research in this area should focus on how HAGL repair affects postoperative shoulder ROM within a clinical setting to improve our understanding of how HAGL repair contributes to shoulder stability.

We observed that patients with HAGL lesions underwent open procedures more frequently compared with patients without HAGL lesions, with just under 50% of HAGL patients undergoing open HAGL repair. The cause of these findings is multifactorial but may be related to historical open repair techniques and technical challenges in performing all-arthroscopic HAGL repair, including poor visualization of the IGHL origin and possible injury to the axillary neurovascular bundle during anchor placement.^9^ While there are publications reporting successful shoulder stabilization utilizing both open and arthroscopic HAGL repair techniques,^1,10,17^ the data surrounding this topic are often limited to small case series with variable follow-ups and thus warrant further investigation with larger long-term studies.

Our data highlight the difficulties in detecting HAGL lesions through preoperative history, physical examination, PROs, and advanced imaging. Additionally, no current guidelines or recommendations are available to help identify these challenging lesions both preoperatively and intraoperatively. While the use of MR arthrogram may improve our ability to detect HAGL lesions,^19^ surgeons who treat patients with shoulder instability should always have a high index of suspicion that a HAGL lesion may be present at the time of surgery and should perform thorough reproducible arthroscopic examinations to avoid overlooking this rare yet clinically important finding.

### Limitations

The findings of this study should be interpreted within the context of its limitations. There was a small number of patients with diagnosed HAGL lesions within our cohort. Thus, the study may not have been adequately powered to detect differences in all examined variables. The low prevalence of HAGL lesions in our study may be because of the difficulty in properly identifying these lesions via arthroscopy. However, our sample size was limited by the data available from the MOON Shoulder Instability database, and the prevalence of HAGL lesions in our study was comparable to rates reported in more recent literature. The prevalence of HAGL lesions in our cohort may have also been lower—given that no patients undergoing revision surgery were included in our cohort. We were unable to calculate the positive predictive value and negative predictive value of other intra-articular pathologies such as Hill-Sachs or anterior labral tears, as our dataset did not include whether they were present on diagnostic arthroscopy. Although we were given data on intraoperative findings, database limitations prevented us from examining more specific factors such as insurance status, use of standard MRI versus MR arthrogram, HAGL lesion size, labral tear length, bone loss, presence of cartilage injury, the frequency of arthroscopic HAGL repair, or postoperative PROs. Like all database studies, the results of our study were also subject to coding inaccuracies and missing data points, which may have influenced our findings. Given the relative paucity of the literature on postoperative outcomes after HAGL repair, it will be important to publish the short- and long-term results of current surgical repair techniques and rehabilitation protocols.

## Conclusion

Approximately one-third of HAGL lesions may be missed on preoperative MRI. Patients with a HAGL lesion were less likely to exhibit classic preoperative imaging findings associated with anterior shoulder instability, such as Hill-Sachs lesions or anterior labral pathology. These patients underwent open stabilization procedures more frequently than patients without HAGL lesions. Given that standard preoperative history, physical examination, and MRI cannot reliably detect HAGL lesions, a thorough arthroscopic examination should be routinely performed when patients undergo operative management for shoulder instability.

## Authors

Ryan D. Freshman, MD (Department of Orthopedic Surgery, University of California–San Francisco, San Francisco, California, USA); Alan L. Zhang, MD (Department of Orthopedic Surgery, University of California–San Francisco, San Francisco, California, USA); C. Benjamin Ma, MD, (Department of Orthopedic Surgery, University of California–San Francisco, San Francisco, California, USA); Brian T. Feeley, MD (Department of Orthopedic Surgery, University of California–San Francisco, San Francisco, California, USA); Shannon Ortiz, MPH (University of Iowa, Iowa City, Iowa, USA); Jhillika Patel, BA (Brigham and Women’s Hospital, Boston, Massachusetts, USA); Warren Dunn, MD (Fondren Orthopedic Group, Houston, Texas, USA); Brian R. Wolf, MD (Department of Orthopedic Surgery, University of California–San Francisco, San Francisco, California, USA); Carolyn Hettrich, MD (Brigham and Women’s Hospital, Boston, Massachusetts, USA); Drew Lansdown, MD (Department of Orthopedic Surgery, University of California–San Francisco, San Francisco, California, USA); and MOON Shoulder Group collaborators: Keith M. Baumgarten, MD (Orthopedic Institute of Sioux Falls, Sioux Falls, South Dakota, USA); Julie Y. Bishop, MD (The Ohio State University Wexner Medical Center, Columbus, Ohio, USA); Matthew J. Bollier, MD (University of Iowa, Iowa City, Iowa, USA); Robert H. Brophy, MD (Washington University, St Louis, Missouri, USA); Jonathan T. Bravman, MD (University of Colorado School of Medicine, Aurora, Colorado, USA); Charles L. Cox, MD, MPH (Vanderbilt University Medical Center, Nashville, Tennessee, USA); Gregory L. Cvetanovich, MD (The Ohio State University Wexner Medical Center, Columbus, Ohio, USA); John A. Grant, MD, PhD (MedSport, University of Michigan, Ann Arbor, Michigan, USA); Rachel M. Frank, MD (University of Colorado School of Medicine, Aurora, Colorado, USA); Grant L. Jones, MD (The Ohio State University Wexner Medical Center, Columbus, Ohio, USA); John E. Kuhn, MD (Vanderbilt University Medical Center, Nashville, Tennessee, USA); Scott D. Mair, MD (University of Kentucky, Lexington, Kentucky, USA); Robert G. Marx, MD, MSc (Hospital for Special Surgery, New York, New York, USA); Eric C. McCarty, MD (University of Colorado School of Medicine, Aurora, Colorado, USA); Bruce S. Miller, MD (MedSport, University of Michigan, Ann Arbor, Michigan, USA); Adam J. Seidl, MD (University of Colorado School of Medicine, Aurora, Colorado, USA); Matthew V. Smith, MD (Washington University, St Louis, Missouri, USA); Rick W. Wright, MD (Vanderbilt University Medical Center, Nashville, Tennessee, USA).

## Supplemental Material

sj-pdf-1-ojs-10.1177_23259671231206757 – Supplemental material for Factors Associated With Humeral Avulsion of Glenohumeral Ligament Lesions in Patients With Anterior Shoulder Instability: An Analysis of the MOON Shoulder Instability Cohort Click here for additional data file. Supplemental material, sj-pdf-1-ojs-10.1177_23259671231206757 for Factors Associated With Humeral Avulsion of Glenohumeral Ligament Lesions in Patients With Anterior Shoulder Instability: An Analysis of the MOON Shoulder Instability Cohort by Ryan D. Freshman, Alan L. Zhang, C. Benjamin Ma, Brian T. Feeley, Shannon Ortiz, Jhillika Patel, Warren Dunn, Brian R. Wolf, Carolyn Hettrich, Drew Lansdown, Keith M. Baumgarten, Julie Y. Bishop, Matthew J. Bollier, Robert H. Brophy, Jonathan T. Bravman, Charles L. Cox, Gregory L. Cvetanovich, John A. Grant, Rachel M. Frank, Grant L. Jones, John E. Kuhn, Scott D. Mair, Robert G. Marx, Eric C. McCarty, Bruce S. Miller, Adam J. Seidl, Matthew V. Smith and Rick W. Wright in Orthopaedic Journal of Sports Medicine

## Appendix

**Table A1:** Prevalence of Preoperative MRI Findings^*a*^

	HAGL	No HAGL	*P*
Anterior glenoid bone loss	4.8	15.1	.35
Hill-Sachs lesion	28.6	54.7	.03^*b*^
Anterior labral tear	66.7	87.2	.01^*b*^
SLAP tear	14.3	15.7	≥.99
HAGL lesion	71.4	0.8	≤.01^*c*^
Rotator cuff tear	0	1.4	≥.99
Biceps pathology	0	1	≥.99

a Data are reported as percentages. HAGL, humeral avulsion of the glenohumeral ligaments; MRI, magnetic resonance imaging; SLAP, superior labrum anterior and posterior.

b *P* < .05.

c *P*< .001.

**Table A2:** Procedures Performed^*a*^

	HAGL	No HAGL	*P*
Any arthroscopic procedure	71.4	97.7	≤.01^*b*^
Arthroscopic Bankart repair	57.1	90.7	≤.01^*b*^
Arthroscopic SLAP repair	9.5	22.1	.28
Arthroscopic debridement	4.8	12.6	.50
Arthroscopic suture plication	4.7	5.7	≥.99
Any open procedure	66.7	7	≤.01^*b*^
Open Bristow-Latarjet	0	4.1	≥.99
Open Bankart repair	23.8	1.4	≤.01^*b*^
Open inferior capsular shift	23.8	2.2	≤.01^*b*^
Open HAGL repair	42.9	1.6	≤.01^*b*^

a Data are reported as percentages. HAGL, humeral avulsion of the glenohumeral ligaments; SLAP, superior labrum anterior and posterior.

b *P* < .001.
