# Supplementary material for: Factors Associated With Humeral Avulsion of Glenohumeral Ligament Lesions in Patients With Anterior Shoulder Instability: An Analysis of the MOON Shoulder Instability Cohort
Source: Orthop J Sports Med. 2023 Oct 27;11(10):23259671231206757. doi: 10.1177/23259671231206757 (PMC10612462; doi:10.1177/23259671231206757)
Supplement: sj-pdf-1-ojs-10.1177_23259671231206757 – Supplemental material for Factors Associated With Humeral Avulsion of Glenohumeral Ligament Lesions in Patients With Anterior Shoulder Instability: An Analysis of the MOON Shoulder Instability Cohort [file sj-pdf-1-ojs-10.1177_23259671231206757.pdf]

One or more of the named authors has declared the following potential conflicts of interest or source of funding:

A.L.Z. has received consulting fees from Stryker and Depuy Mitek, research support from Zimmer, and hospitality payments from Arthrex. C.B.M. has received consulting fees from Linvatec, Zimmer Biomet, Wright Medical, Medacta USA, and Stryker; royalties from Linvatec; hospitality payments from Arthrex; and research support from Aesculap. B.T.F. has received consulting fees from Kaliber, educational fees from Evolution Surgical, hospitality payments from Zimmer Biomet, and is on the board of directors for Bioniks. B.R.W. has received royalties and educational/grant support from ConMed, education payments from Wardlow Enterprises, speaking fees and educational grant/support from Smith & Nephew, speaking fees and educational grant/support from Arthrex/Wardlow, consulting fees and speaking fees from Linvatec, is on the advisory board for and receives personal fees from UnitedHealth Care, and is the founder/owner of SportsMed Innovate. C.H. has received hospitality payments from Tornier, Zimmer Biomet, and Wright Medical. D.L. has received grant and educational research support from Arthrex; education payments from Evolution Surgical, Medwest, and Smith & Nephew; hospitality payments from Wright Medical; and consulting fees from Vericel and Allosource.

Contributing authors have the following disclosures:

K.M.B. has received speaking fees from Wright Medical and consulting fees from Miach, Stryker, Wright Medical, and Arthrex. J.Y.B. has received consulting fees from Stryker and Linvatec and speaking fees from Smith & Nephew. J.T.B. has received consulting fees from Smith & Nephew, DJO Global, and Encore Medical, speaking fees from Smith & Nephew, and royalties from Shukla Medical. M.J.B. has received education payments from Wardlow Enterprises and hospitality payments from Stryker. R.H.B. receives consulting and speaking fees from Arthrex and Sanofi and educational payments from Arthrex. G.L.C. has received consulting fees from Smith & Nephew, grant support from Arthrex, and educational support from Medwest. R.M.F. has received consulting fees from Arthrex, JRF, and AlloSource; research support from Arthrex and Smith & Nephew; and education payments from Gemini Mountain Medical and Pinnacle. J.A.G. has received consulting fees from Vericel and JRF, educational fees from Pinnacle, hospitality fees from Smith & Nephew, personal fees from JRF Ortho and ConMed, and grant support from JRF Ortho, Arthrex, and Aesculap. G.L.J. has received honoraria from MTF and educational payments from CDC Medical. S.D.M. has received educational payments from Zimmer Biomet, speaking fees from Smith and Nephew, fellowship support from Arthrex and Smith & Nephew, and consulting fees from Smith & Nephew and Heron Therapeutics. R.G.M. has received publishing royalties from Springer and Demos Health and is on the science advisory board at MEND Nutrition. E.C.M. has received consulting fees from Zimmer Biomet and Flexion Therapeutics; educational payments from Smith & Nephew, Arthrex, Mitek, and Ossur; consulting fees from Medical Device Business Services and Zimmer Biomet; speaking fees from Arthrex; and royalties from Zimmer Biomet. B.S.M. has received consulting fees from Arthrex and FH Orthopedics, royalties from FH Orthopedics, hospitality payments from Arthrex, and education payments from Pinnacle. A.J.S. has received consulting fees from DJO and Medacta, research support from Smith & Nephew; honoraria from Encore Medical, and education payments from Arthrex. M.V.S. has received educational payments from Arthrex and grant support from DJO. R.W.W. has received personal fees from Wolters Kluwer–Lippincott Williams & Wilkins, royalties and stocks from Responsive Arthroscopy, royalties from MiCare, and stock options from Hyalex.
